# Comparison of the short-term results of nasal and temporal 180° selective laser trabeculoplasties for open-angle glaucoma

**DOI:** 10.5935/0004-2749.20230044

**Published:** 2023

**Authors:** Jayter Silva Paula, Cassia Senger, Carlos Gustavo de Moraes

**Affiliations:** 1 Department of Ophthalmology, Faculdade de Medicina de Ribeirão Preto, Universidade de São Paulo, Ribeirão Preto, SP, Brazil.; 2 Bernard and Shirlee Brown Glaucoma Research Laboratory, Department of Ophthalmology, Edward S. Harkness Eye Institute, Columbia University Medical Center, New York, NY, USA.

**Keywords:** Glaucoma, open-angle, Laser therapy/methods, Intraocular pressure, Trabeculectomy/methods, Glaucoma de ângulo aberto, Terapia a laser/mé­todos, Pressão intraocular, Trabeculoplastia/métodos

## Abstract

**Purpose:**

The nasal sector of the anterior chamber angle may present a higher density of collector channels, which may influence the results of angle surgeries. Considering the anatomical differences in the anterior chamber angle, we compared the results of the nasal and temporal 180° selective laser trabeculoplasty approaches for open-angle glaucoma.

**Methods:**

A retrospective chart review was conducted for patients with open-angle glaucoma (primary, pseudoexfoliation, and pigmentary) who underwent at least one 180° selective laser trabeculoplasty session between December 2016 and October 2018. The nasal (N1) or temporal (T1) sectors were chosen at the physician’s discretion. Patients who did not experience decreased intraocular pressure between 3 and 6 months again underwent 180° selective laser trabeculoplasty in the opposite angle sector (T2 and N2). The main outcome measured was decrease in intraocular pressure at 6-month follow-up, after the last selective laser trabeculoplasty. A multivariable regression analysis was used to evaluate factors associated with decreased intraocular pressure after treatment.

**Results:**

The procedure was performed initially in 45 eyes (N1, 25; T1, 20 eyes) and repeated in the opposite anterior chamber angle sector in 19 eyes (N2, 11; T2, 8 eyes). Analysis of variance revealed that only the N1 approach presented a significant difference in the decrease in intraocular pressure as compared with the T1, N2, and T2 approaches (p=0.0014). The baseline intraocular pressure (p=0.021) and anterior chamber angle sector (N1; p=0.044) correlated with decreased intraocular pressure.

**Conclusion:**

Compared with the temporal approach, 180° selective laser trabeculoplasty performed initially in the nasal sector was associated with a more significant decrease in intraocular pressure. Considering the sectorial differences in the anterior chamber angle, further prospective trials are warranted to confirm our findings and provide more-efficient selective laser trabeculoplasty protocols.

## Abstract (pt)

**Objetivo:**

O setor nasal do ângulo da câmara anterior pode apresentar maior densidade de canais coletores, o que pode influenciar no resultado de cirurgias angulares. Considerando as diferenças anatômicas no ângulo da câmara anterior, comparamos os resultados das abordagens de trabeculoplastia seletiva a laser nasal e temporal de 180 graus no glaucoma de ângulo aberto.

**Métodos:**

Revisão retrospectiva de prontuários de pacientes com glaucoma de ângulo aberto (primária, pseudoexfoliação e pigmentar), que realizaram pelo menos uma sessão de trabeculoplastia seletiva a laser de 180 graus entre dezembro/2016 e outubro/2018. O setor nasal (N1) ou temporal (T1) foi escolhido a critério do médico. Os pacientes que não apresentaram diminuição da pressão intraocular (PIO) entre 3 e 6 meses foram retratados com trabeculoplastia seletiva a laser de 180 graus no setor de ângulo oposto (T2 e N2). O principal resultado medido foi a diminuição da pressão intraocular no 6º mês de acompanhamento após a última trabeculoplastia seletiva a laser. Uma análise de regressão multivariável avaliou os fatores associados à redução da pressão intraocular após o tratamento.

**Resultados:**

O procedimento foi realizado inicialmente em 45 olhos (N1=25, T1=20 olhos), e repetido no setor ângulo da câmara anterior oposto em 19 olhos (N2 = 11, T2 = 8 olhos). Os testes ANOVA mostraram que apenas a abordagem N1 apresentou diferença significativa na diminuição da pressão intraocular em relação a T1, N2 e T2 (p=0,0014). A pressão intraocular basal (p=0,021) e o setor ângulo da câmara anterior (N1; p=0,044) se correlacionaram com a diminuição da pressão intraocular.

**Conclusão:**

A trabeculoplastia seletiva a laser de 180 graus realizado inicialmente no setor nasal foi associado a uma diminuição mais significativa da pressão intraocular em comparação com a abordagem temporal. Considerando as diferenças setoriais no ângulo da câmara anterior, mais estudos prospectivos são necessários para confirmar nossos achados e fornecer protocolos para trabeculoplastia seletiva a laser mais eficientes.

## INTRODUCTION

Laser trabeculoplasty modalities are noninvasive therapies aimed at reducing intraocular pressure (IOP) in patients with glaucoma. They presented mid- and long-term effects comparable with those of topical medications regarding both IOP control^(1-4)^ and visual field progression in multicenter trials involving patients with open-angle glaucoma (OAG)^(5)^.

Selective laser trabeculoplasty (SLT) is believed to selectively target the pigmented trabecular meshwork cells without causing thermal or collateral damage to the neighboring cells or matrix^(1-3)^. SLT has been proven as a safe, well-tolerated, and effective therapy for IOP reduction in several types of glaucoma^(3,5-12)^. Owing to its very short pulse duration compared with the thermal relaxation time of the local tissues, the adjacent regions do not absorb the laser energy; thus, the spread of heat damage is minimized. The significantly lower anterior chamber angle (ACA) tissue disruption than that with the argon laser allows for more SLT retreatment sessions^(4-6)^. SLT can be considered as a primary treatment option in patients who are intolerant of or non-adherent to glaucoma medications, without interfering with the success of future surgeries^(6)^.

In a SLT session, both the selection of the ACA region and extension of treatment are mostly decided in the discretion of glaucoma specialists rather than based on the current anatomical knowledge of the aqueous humor outflow system. Circumferential differences in the structure of Schlemm’s canal and the distribution of collector channels (CCs) have been implied in the discrepant results of minimally invasive glaucoma surgery (MIGS)^(7,8)^. Considering the circumferential anatomical differences in the aqueous humor drainage system, the purpose of our study was to compare results between the nasal and temporal 180° SLT treatment approaches in patients with OAG.

## METHODS

### Study design

This retrospective study included all patients with primary OAG, pigmentary glaucoma, or pseudoexfoliation glaucoma who underwent a 180° SLT treatment at the University of São Paulo Medical School (Ribeirão Preto, Brazil). The patients were >18 years of age and had no previous history of either laser treatment or intraocular surgery, except for non-complicated cataract surgery (performed >6 months before SLT). A comprehensive ophthalmological examination had been previously performed, and all the patients included in the analysis presented glaucomatous optic neuropathy defined on clinical examination, pre-SLT IOP >21 mmHg measured anytime (at least twice) with or without medication, open angles with moderate to intense trabecular pigmentation, and mild to moderate visual field defects (24-2 standard mean deviation better than the Swedish interactive thresholding algorithm standard of 12 dB). The laser procedures were performed between December 2016 and October 2018 in the patients with no adequate IOP control (low treatment compliance, substantial adverse effects with eyedrops, and at least 2 IOP measurements higher than the individualized target IOP).

The study was conducted in accordance with the tenets of the Declaration of Helsinki. Ethics approval for the use of the patients’ retrospective data was obtained from the Ribeirão Preto Clinical Hospital Ethics Committee (2019/4.048.791).

### Laser treatments

SLT was performed using LIGHTLas SLT Deux (400-µm spot size, 3-ns duration; LightMed Corporation, CA, USA) and a specific SLT lens with flange (Ocular Latina SLT with Flage, Ocular Instruments, WA, USA). The laser energy levels ranged from 0.3 to 1.2 mJ per pulse, adjusted after trabecular meshwork (TM) pigmentation in 0.1-mJ increments. If no cavitation bubble was observed at the initial energy level, the energy was increased to the lowest possible level to produce “champagne” bubbles. During the laser treatment, formation of large bubbles was monitored, and the energy level was titrated downward as necessary. For the 180° treatment, 40-50 spots were performed for the treatment of either the nasal or temporal sector.

One experienced ophthalmologist (JSP) performed the laser procedures in all the patients consecutively. The nasal (N1) or temporal (T1) sectors were first chosen in the ophthalmologist’s best discretion. The IOP measurements were scheduled in the morning hours (8:00 to 11:00 AM) during each of the following visits: at baseline (immediately before the procedure) and after treatment, that is, after 10 days, 40 days, 2 months, and 6 months. During each visit, two consecutive Goldmann applanation tonometry measurements were obtained per eye, and the average of these values were considered for the statistical analysis.

Patients who presented IOP reduction <3 mmHg between 3 and 6 months after the first SLT session, attributed as an unsuccessful result, were promptly treated again with 180° SLT in the opposite angle sector (T2 and N2). A single 0.2% brimonidine drop was applied immediately after treatment, and 1% dexamethasone was prescribed twice a day for 7 days. The patients were oriented to continue their current antiglaucomatous medication until a clinical indication for therapy reduction based on the IOP reduction was determined.

### Statistical analysis

The primary outcome measured was the decrease in absolute IOP at 6-month follow-up from the last laser procedure. Continuous variables are described as mean and standard deviation. Data were analyzed using analysis of variance (ANOVA) with Bonferroni’s posttest correction. Variables with abnormal distributions were compared using nonparametric tests. Multivariate regression with mixed-effects models (adjusted for the inclusion of both eyes of the same patient) was also performed using age, diagnosis, the baseline IOP, the number of eye drops applied, and the angle sector treated as covariables. The analyses were performed using commercially available software (Stata version 14; StataCorp LP, College Station, TX). Statistical signifi­cance was defined at p<0.05.

## RESULTS

Forty-five eyes of 31 patients (19 men and 12 women), aged 56.3 ± 10.8 years, were treated with at least one SLT session. The distribution of the treated eyes according to the diagnosis was as follows: primary OAG, 33 eyes (25 patients); pigmentary glaucoma, 8 eyes (4 patients); and pseudoexfoliation glaucoma, 4 eyes (2 patients). The preoperative visual field mean deviation was-6.2 ± 2.8 dB. A significant reduction in IOP was observed between the baseline and the last follow-up (10.2 ± 1.1 months; 18.0 ± 2.8 mmHg vs 14.9 ± 2.3 mmHg; p<0.001, by the Wilcoxon test). Although 14 eyes had reduced medical treatment after SLT, no significant differences were observed in the number of antiglaucomatous eye drops applied (1.8 ± 0.8 vs 1.5 ± 0.9; p=0.159) and the mean baseline IOP between N1 and T1 (18.3 ± 3.3 mmHg vs 18.0 ± 1.7 mmHg; p=0.98; Table 1). The distribution of the IOP results is presented in table 2.

**Table 1:** Demographic and clinical characteristics of the patients who underwent the initial nasal (N1) or temporal (T1) 180° selective laser trabeculoplasty (SLT) approaches

	Nasal first (N1)	Temporal first (T1)	p Value
Number of eyes (patients)	25 (17)	20 (14)	--
Female (%)	6 (35.3)	6 (42.9)	0.72*
Age, years	53.8 ± 11.6	57.5 ± 10.1	0.52**
Glaucoma diagnosis, no. of eyes			0,90***
Primary open-angle glaucoma	19	14	
Pigmentary glaucoma	4	4	
Pseudoexfoliation glaucoma	2	2	
Baseline IOP, mmHg	18.3 ± 3.3	18.0 ± 1.7	0.98**
Number of rye drops	1.8 ± 0.8	1.5 ± 0.9	0.16**
24-2 VF mean deviation, dB	-7.1 ± 3.6	-5.4 ± 2.1	0.19**
Mean RNFL, mm	85.9 ± 10.2	92.5 ± 19.7	0.52**

IOP= intraocular pressure; VF= visual field; RNFL= retinal nerve fiber layer.

* Fisher exact test.

** Mann-Whitney *U* test.

*** Chi-square test.

**Table 2:** Intraocular pressures in the eyes treated with 180° selective laser trabeculoplasty (SLT) initially with nasal (N1) or temporal (T1) approaches and again treated in the opposite nasal (N2) or temporal (T2) angle sectors

	Intraocular Pressure (mmHg)			
	Nasal-first (N1, n=17)	Temporal-first (T1, n=9)	Nasal-second (N2, n=11)	Temporal-second (T2, n=8)
Baseline	18.3 ± 3.3	18.0 ± 1.7	17.5 ± 1.6	17.1 ± 3.3
10 days	15.5 ± 2.8	16.2 ± 2.1	17.1 ± 2.0	16.0 ± 2.9
40 days	13.0 ± 3.9	14.1 ± 2.2	16.2 ± 2.8	14.5 ± 3.0
60 days	12.9 ± 3.1	13.9 ± 1.9	15.8 ± 1.9	15.3 ± 2.2
6 months	13.4 ± 2.4	14.6 ± 1.5	14.1 ± 1.3	14.0 ± 3.4

Data are presented as mean ± standard deviation.

**Figure 1:**
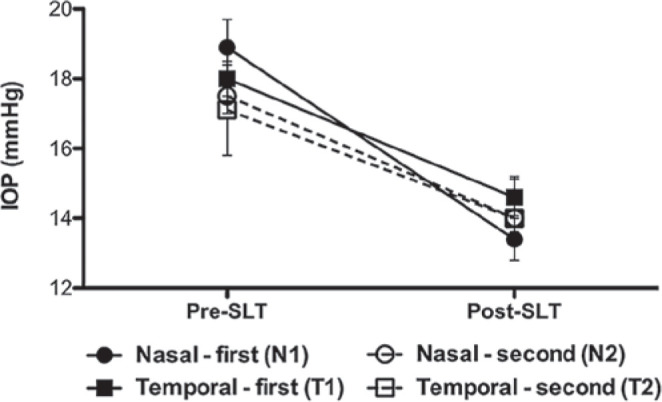
Intraocular pressure (IOP) changes observed before and after 180° SLT performed in different angle sectors (N1: nasal first; T1: temporal first; N2: repeated nasal; T2: repeated temporal). Data points and error bars represent mean ± standard error.

The procedure was performed initially in the nasal or temporal sector in 25 and 20 eyes, respectively, and repeated in the opposite angle sector in 19 eyes (nasal, 11 eyes [58%]; temporal, 8 eyes 42%]). The ANOVA revealed that only the N1 approach (-5.5 ± 1.4 mmHg) presented a significant difference in the decrease in IOP compared with the other approaches (T1=-3.5 ± 1.0 mmHg; N2=-3.4 ± 0.9 mmHg; T2=-3.1 ± 0.9 mmHg; p=0.0014; Figure 1). After the multivariate regression analysis, only baseline IOP (p=0.021) and angle sector (N1; p=0.044) correlated with decreased IOP (Table 3).

**Table 3:** Multivariable analysis with mixed-effects model of factors associated with decreased intraocular pressure (IOP) in patients who underwent 180° selective laser trabeculoplasty (SLT)

IOP difference	Coefficient	SE	p Value	[95% CI]	
Sex (female)	-0.473	0.491	0.336	-1.437	0.490
Age	0.036	0.023	0.124	-0.010	0.083
Diagnosis					
Pigmentary glaucoma	-0.040	0.939	0.965	-1.882	1.800
Pseudoexfoliation glaucoma	-0.707	0.849	0.405	-2.373	0.958
Angle Sector					
Temporal first	-1.745	0.617	**0.005**	-2.955	-0.534
Repeated nasal	-1.772	0.627	**0.005**	-3.003	-0.542
Repeated temporal	-1.854	0.604	**0.002**	-3.039	-0.669
Baseline IOP	0.200	0.087	**0.022**	0.029	0.371
Number of eye drops					
1	-0.091	1.227	0.941	-2.498	2.314
2	-0.348	1.184	0.768	-2.670	1.972
3	-0.371	1.196	0.756	-2.716	1.973
Constant	0.021	2.1240	0.992	-4.142	4.185

SE= Standard error; 95% CI= 95% confidence interva.

## DISCUSSION

In this study, we investigated the IOP reduction obtained with two different approaches of SLT treatment in the ACA regions (i.e., nasal vs temporal 180° sectors). Notwithstanding the correlation between the baseline IOP and SLT results, when the nasal sector was the first SLT region treated (N1 approach), a significantly greater reduction in IOP was observed in this series of patients who did not present extremely high initial IOPs. To the best of our knowledge, no previous study has compared results between nasal and temporal SLT 180° approaches.

The mechanism of action of the SLT for lowering IOP is not entirely understood and is likely multifactorial.^(9)^ Photothermolysis selectively targeting the pigmented TM cells after SLT may be the initial factor in the cascade of events culminating in the lower resistance in the aqueous humor outflow system^(10,11)^.

A comparative study of 180° and 360° SLT approaches showed the superiority of 360° SLT treatment in reducing IOP in Japanese patients with OAG^(12)^. This favo­rable result of 360° SLT was also described by Prasad et al., who also showed smaller IOP fluctuations up to 2 years of follow-up^(13)^.

In a report that examined the mechanism of action of SLT in TM cells in regulating the permeability of endothelial cells in Schlemm’s canal, the cytokines released showed increased macrophage activity in the TM, which could explain the better SLT results observed with the 360° SLT treatment; that is, a more extensive area may lead to more endothelial cells producing higher levels of outflow-upregulating mediators^(6)^.

Nevertheless, in a systematic review of the effect of SLT on IOP control, the 360° SLT approach was comparable with medical treatment, and no significant difference was found between the 360° and 180° SLT approaches^(10)^.

Regarding the comparison between the 90° and the 180° protocols for SLT, no definitive conclusion could be derived regarding the difference in IOP reduction^(14,15)^. Smaller ACA areas of treatment (90°) would induce both lower overall inflammatory reaction and less release of mediators, which explains the worse results in patients who underwent the 90° SLT treatment in some studies^(16,17)^. However, such rationale did not explain the equivalent results observed in other comparative studies^(12,16)^, which may suggest that other unknown variations in the laser techniques, such as details on the sectors of the ACA chosen for treatment, influenced the lack of significant difference between the SLT approaches^(18)^. We speculate that the circumferential location in the ACA rather than the extension of laser treatment may impact the results of SLT. Studies involving the histological evaluation of the ACA have found higher numbers of CCs, with significantly higher mean cross-sectional CC areas, in the nasal than in the temporal region^(7)^. CCs usually leave Schlemm’s canal diagonally or at right angles and vary in shape, size, and number (20 to 30 CCs circumferentially distributed around the globe)^(7,19)^. By using finite element models, Martínez Sánchez et al.^(20)^ showed that the position and opening of the CCs influenced the IOP significantly. Cha^(8)^ observed a lower perfusion outflow resistance in the ACA sectors with expanded TM tissue and a higher number of CCs, particularly in the nasal (41.2%) and lower quadrants (31.7%) than in the temporal (14.2%) and upper quadrants (12.6%). These CC features in the nasal ACA sector might be considered as a significant factor influencing the results of MIGS^(15,21-23)^. Moreover, an ACA expansion after SLT was recently reported in eyes successfully treated with SLT^(24,25)^. Such ACA widening, with a probable opening of Schlemm’s canal and CCs, should be considered as an additional structural factor implicated in the SLT results.

Repeated SLT treatment has been reported to result in worse IOP reduction than the first laser treatment in cases of OAG, which might explain the worse results in N2 than in N1^(9,14,15)^. Our choice of treating the non-overlapping sectors was then based on the results of George et al.^(14)^, who reported better IOP results with the 180° non-overlapping repeated SLT approach. Altogether, the findings of better IOP reduction after the first rather than the later SLT sessions, the SLT ACA-widening effect, and the anatomical ACA differences, particularly the nasal CC features impacting the IOP distinctly, may help understand the better SLT results observed in our study.

Finally, consonant with earlier reports^(11,14,26,27)^, we found a positive correlation between baseline IOP and IOP reduction^(11,27,28)^. High baseline IOP has been shown to be a predictor of SLT success, even in patients with normotensive glaucoma^(9)^.

Even considering the significant results observed, we must recognize that our study has limitations such as the relatively small sample size, the inclusion of both phakic and pseudophakic patients and only two treatment-naive patients, the short follow-up period, the retrospective design, the absence of comparator groups with results from eyes treated with either eye drops or the 360° approach, and the lack of information on the individual distribution of CCs among the treated eyes.

Despite these limitations, our results showed that the 180° SLT performed initially in the nasal sector was associated with higher IOP reduction than the temporal sector laser approach. Considering all the sectorial diffe­rences in the circumferential aqueous humor outflow system^(21,22)^, further prospective randomized clinical trials are warranted to assess our findings and evaluate the use of aqueous angiography imaging prior to MIGS and SLT. Ultimately, customization of the ACA for SLT, as currently proposed for MIGS, could improve IOP-related outcomes when coupled with imaging of the outflow pathways.
